# Serum (1→3)-β-D-glucan and galactomannan levels in patients with cystic fibrosis: a retrospective cohort study

**DOI:** 10.1186/s12890-018-0614-8

**Published:** 2018-03-27

**Authors:** Johannes Träger, Volker Otto Melichar, Renate Meyer, Manfred Rauh, Christian Bogdan, Jürgen Held

**Affiliations:** 1 Mikrobiologisches Institut - Klinische Mikrobiologie, Immunologie und Hygiene, Universitätsklinikum Erlangen und Friedrich-Alexander-Universität (FAU) Erlangen-Nürnberg, Wasserturmstr. 3/5, 91054 Erlangen, Germany; 2 Kinder- und Jugendklinik, Universitätsklinikum Erlangen und Friedrich-Alexander-Universität (FAU) Erlangen-Nürnberg, Erlangen, Germany

**Keywords:** (1→3)-β-D-glucan, Galactomannan, Biomarker, Cystic fibrosis, *Aspergillus*, Allergic bronchopulmonary aspergillosis, ABPA, Aspergillosis, Lung function, FEV1

## Abstract

**Background:**

*Aspergillus fumigatus* is frequently encountered in sputum samples of patients with cystic fibrosis (CF), which traditionally has been interpreted as saprophytic airway colonization. However, this mere bystander role has been challenged by recent data. There is now evidence that *Aspergillus fumigatus* accelerates the decline of pulmonary function.

(1→3)-β-D-glucan (BDG) and galactomannan (GM) are highly sensitive fungal biomarkers that are used to diagnose invasive fungal disease. However, their diagnostic value in CF patients is largely unknown.

**Methods:**

We conducted a retrospective cohort study on 104 CF patients to determine whether serum BDG and GM levels correlate with parameters such as *Aspergillus-*positive sputum cultures and lung function.

**Results:**

*Aspergillus fumigatus* was persistently detected in 22 of the 104 CF patients (21%). Mean serum BDG and GM levels in the *Aspergillus*-positive patients were significantly higher than in those without persistent *Aspergillus* detection (89 versus 40 pg/ml [*p* = 0.022] and 0.30 versus 0.15 ODI [*p* = 0.013], respectively). 27 and 7 patients had elevated BDG (≥ 60 pg/ml) or GM levels (> 0.5 ODI), respectivly. BDG and GM levels showed a significant correlation (*p* = 0.004). Patients with increased serum concentrations of BDG were more frequently *Aspergillus*-positive (40.7 versus 14.3%, p = 0.004) and had a significantly lower forced expiratory volume in one second (FEV1) than patients with a normal BDG (61.6 versus 77.1%, *p* = 0.007). In the multivariate analysis, BDG but not GM or the growth of *A. fumigatus*, proved to be an independent predictor for the FEV1.

**Conclusions:**

CF patients with persistent *Aspergillus* detection have elevated BDG and GM levels which ranged between healthy and invasively infected patients. Serum BDG may be superior to GM and fungal culture in predicting an impaired lung function in CF patients.

## Background

Cystic fibrosis (CF) is the most frequent lethal autosomal recessive disorder in Caucasians. It is caused by mutations in the cystic fibrosis transmembrane conductance regulator (CFTR) gene leading to impaired ion transport across epithelial cells. The disease affects primarily the exocrine glands, resulting in pancreatic insufficiency and progressive pulmonary disease; the latter is the major cause of morbidity and mortality in CF patients. Within the lungs, highly viscous sputum facilitates the colonization and infection of the lower respiratory tract with various microorganisms [1].

While the role of bacterial pathogens, like *Staphylococcus aureus*, *Pseudomonas aeruginosa* or *Burkholderia cepacia* complex, for the progression of the disease is well established, far less is known about the impact of chronic airway colonization with fungi. *Aspergillus fumigatus*, *Scedosporium* species, *Candida albicans* and *Exophiala dermatitidis* are frequently encountered in sputum samples of CF patients [2]. In particular, the traditional view on *A. fumigatus,* which has been regarded as saprophytic microorganism with doubtful clinical significance, is challenged by recent data. There is evidence that chronic *A. fumigatus* colonization is associated with an increased risk of hospitalization for pulmonary exacerbation and lower lung function and that this effect is aggravated by *P. aeruginosa* co-infection [3, 4]. Furthermore, it seems that antifungal therapy is of benefit for patients who are non-responsive to antibiotics and that the administration of itraconazole to *A. fumigatus*-sensitized CF patients can improve lung function [5, 6].

Galactomannan (GM) is a major component of the fungal cell wall. It is primarily detected in the serum or bronchoalveolar lavage fluid specimens of patients with invasive aspergillosis [7]. Another fungal antigen used for this purpose is (1→3)-β-D-glucan (BDG). BDG differs in several aspects from GM. Firstly, it is produced by all medically relevant fungi (e.g. *Candida* spp., *Aspergillus* spp., *Pneumocystis jirovecii*) apart from zygomycetes and *Cryptococcus neoformans* which contain no or only low amounts of BDG in their cell walls [8]. Secondly, BDG has superior sensitivity for diagnosis of invasive aspergillosis compared to serum GM [9].

Only little is known about serum BDG and GM levels in patients with CF. Because of their high sensitivity, BDG and/or GM measurements might help to identify patients with fungus-associated morbidity. Therefore, we initiated a retrospective study in order to analyze causal relationships between clinical and microbiological parameters and BDG- as well as GM-antigenemia.

## Methods

### Study population

We conducted a retrospective cohort study at the University Hospital Erlangen, Germany, a 1400-bed tertiary care hospital. All patients with CF (children, adolescents and adults), who presented to the CF outpatient clinic between September 2015 and October 2016, were enrolled. Patients with a history of lung transplantation were excluded. As part of the routine diagnostic work-up paired respiratory and serum samples were taken.

### Microbiological analyses

Respiratory samples were analyzed for the growth of bacteria (e.g. *S. aureus, P. aeruginosa*) and fungi (e.g. *A. fumigatus*) using Columbia blood agar plates, chocolate agar plates, endo agar plates and Sabouraud dextrose agar plates with 0.5 mg/ml chloramphenicol. If necessary, the samples were pretreated with dithiothreitol at a final concentration of 50 μg/ml. The agar plates were then incubated for at least seven days at 37 °C in ambient air with 5% CO_2_ for the culture of bacteria and at 28 °C in ambient air for the culture of fungi. Relevant microorganisms were differentiated to the species level by Matrix Assisted Laser Desorption Ionization - Time of Flight Mass Spectrometry (MALDI-TOF-MS; Bruker Daltonik GmbH, Germany). Filamentous fungi were differentiated by microscopy of a lactophenol cotton blue wet mount preparation.

Serum samples were tested for *Aspergillus*-specific antibodies (Aspergillose Fumouze, Fumouze Diagnostics, France), *Aspergillus*-specific IgE, recombinant *Aspergillus* antigens f4 and f6 [rAsp f4 IgE, rAsp f6 IgE] (ImmunoCAP 250, Thermo Fisher Scientific, Sweden) and IgG against alkaline protease, elastase and exotoxin A of *P. aeruginosa* (Mediagnost GmbH, Germany). The sera were then stored at − 20 °C. In October 2016, the stored samples were tested in batch for their content of BDG (Fungitell^®^ assay; Associates of Cape Cod, USA) and GM (Platelia™ *Aspergillus* Ag assay; Bio-Rad, France). Both assays were performed according to the manufacturer’s instructions. BDG levels above the upper validation limit were diluted and retested. BDG levels below the lower validation limit were calculated by extrapolation. In July 2017, the same serum samples were tested for human fatty acid binding protein 2 (FABP2) using the Quantikine ELISA human FABP2/I-FABP kit (R&D Systems Europe, UK) according to the manufacturer’s instructions. The use of these sera was approved by the local ethics committee (application number 7-17B).

### Clinical data and classification of patients

Demographic data and clinical information (body mass index [BMI], cough frequency, sputum production, annual number of pulmonary exacerbations, anti-infective therapy, corticosteroid use, exocrine pancreatic insufficiency, CF-related *Diabetes mellitus* [CFRD], CF-related liver disease [CFLD], forced expiratory volume in 1 s [FEV1_predicted_] at serum sampling, and immunological results (C-reactive protein [CRP], leucocyte count [WBC], total serum IgG and IgE) were obtained from all patients analyzed. Following the Global Initiative for Chronic Obstructive Lung Disease (GOLD) classification of severity of airflow obstruction, the study population was divided into patients with mild or no airflow limitation (FEV1_predicted_ ≥ 80%) and patients with moderate to very severe airflow limitation (FEV1_predicted_ < 80%) at the time of serum sampling. Furthermore, on the basis of the Leed’s criteria for chronic *P. aeruginosa* infection [10], patients were classified according to their respiratory culture results during the past two years in persistently colonized (growth of *A. fumigatus* in > 50% of total samples or at least in all of the last three samples before serum sampling), intermittently colonized (detection of *A. fumigatus* that does not fulfil the criteria for persistently colonized patients) and not colonized (no detection of *A. fumigatus*).

### Statistical analysis

Statistical analysis was performed using SPSS-V24 (SPSS Inc., USA) and MedCalc Statistical Software-V16.8 (MedCalc Software bvba, Belgium). Data are given as mean +/− standard deviation (SD) or median with interquartile range (IQR). Receiver-operating-characteristic (ROC)-analysis was used to evaluate the ability of BDG and GM to distinguish between *Aspergillus*-positive and *Aspergillus*-negative patients. The highest Youden index indicated the optimal cut-off. As BDG and GM values were not normally distributed, the chi-square test, the Mann-Whitney-U test and the Spearman coefficient of correlation were used for comparison of variables. Differences were considered significant when *p* values were <  0.05.

## Results

### Study population

The local cystic fibrosis cohort consisted of 137 patients. Archived serum samples were available from 107 patients. Three patients had to be excluded, because BDG-testing repeatedly produced discrepant results (*n* = 2) or a respiratory tract specimen for microbiological analysis was not taken at the day of serum sampling (*n* = 1). Thus, 104 patients were included in the study.

**Fig. 1:**
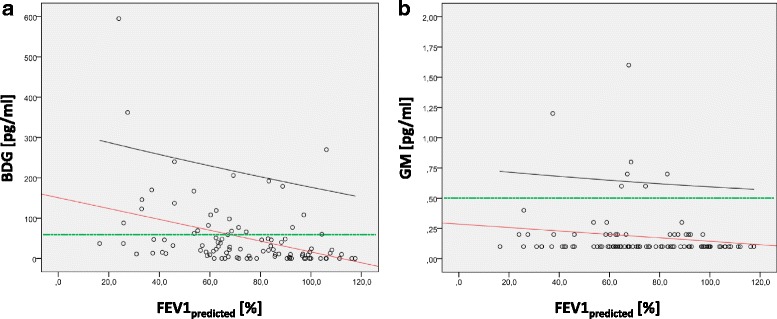
Correlation of the FEV1 at serum sampling with BDG and GM. BDG (**a**.) and GM (**b**.) show a significant correlation with the FEV1 (*p* <  0.001 and 0.037, respectively). The effect size is 0.5 for BDG and 0.2 for GM, respectively. This corresponds to a medium effect for BDG and a small effect for GM according to Cohen [11]. The regression line is depicted in red, the 95%-confidence interval in black and the manufacturer’s cut-off value for invasive fungal disease in green. FEV1: forced expiratory volume in 1 s; BDG: (1→3)-β-D-glucan; GM: galactomannan

Demographic data, clinical and microbiological results for all patients as well as for subgroups (stratified according to FEV1_predicted_ values at the time of serum sampling, the growth of *A. fumigatus* and the concentration of serum BDG and GM) are shown in Tables 1 and 2. Correlations of FEV1_predicted_ values, BDG and GM concentrations with continuous clinical and microbiological parameters are given in Table 3 and Fig. 1.

**Table 1:** Patient demographics, microbiological and clinical results stratified after FEV_1_ at serum sampling and persistent *A. fumigatus* detection

	All patients	FEV_1_ at serum sampling			Persistent *A. fumigatus* detection		
	(*n* = 104)	(< 80% pred.) (*n* = 55)	(≥80% pred.) (*n* = 42)	*P* value	No (*n* = 82)	Yes (*n* = 22)	*P* value
Age [years (min-max)]	19.3 (4–51)	24.0 (6–51)	15.7 (7–31)	< 0.001	17.9 (4–51)	24.8 (10–51)	0.001
Sex [female/male]	43/61	22/33	17/25	0.962	32/50	11/11	0.353
BMI [kg/m^2^ (± STD)]	19.7 (± 3.9)	20.4 (± 3.6)	19.2 (± 3.6)	0.114	19.2 (± 3.7)	21.6 (± 3.8)	0.006
Mean BDG [pg/ml (± STD)]	50 (± 84)	68 (± 100)	32 (± 57)	0.003	40 (± 63)	89 (± 131)	0.022
Median BDG [pg/ml (IQR)]	22 (3–62)	37 (12–82)	11 (0–42)		18 (2–48)	50 (14–115)	
BDG [normal/elevated, (% elevated)]	77/27 (26.0%)	36/19 (34.5%)	36/6 (14.3%)	0.024	66/16 (19.5%)	11/11 (50.0%)	0.004
Mean GM [ODI (± STD)]	0.18 (± 0.22)	0.22 (± 0.28)	0.14 (± 0.10)	0.150	0.15 (± 0.12)	0.30 (± 0.40)	0.013
Median GM [ODI (IQR)]	0.10 (0.1–0.2)	0.10 (0.1–0.2)	0.10 (0.1–0.1)		0.10 (0.1–0.1)	0.10 (0.1–0.3)	
GM [normal/elevated, (% elevated)]	97/7 (6.7%)	49/6 (10.9%)	41/1 (2.4%)	0.108	78/4 (4.9%)	19/3 (13.6%)	0.145
Persistent *A. fumigatus* detection [no. of patients (%)]	22 (21.2%)	16 (29.1%)	6 (14.3%)	0.084	–	–	–
*A. fumigatus*-specific antibodies [normal/elevated, (% elevated)]	100/4 (3.8%)	52/3 (5.5%)	41/1 (2.4%)	0.451	78/4 (4.9%)	22/0 (0%)	0.291
*A. fumigatus*-specific IgE level [kU/l, (± STD)]	3.8 (± 9.9)	5.2 (± 11.3)	2.3 (± 8.2)	0.004	4.5 (± 10.9)	1.1 (± 2.0)	0.687
rAsp f4-IgE level [kUA/l, (± STD)]	0.5 (± 2.5)	0.7 (± 3.1)	0.3 (± 1.5)	0.073	0.6 (± 2.7)	0.1 (± 0.2)	0.879
rAsp f6-IgE level [kUA/l (± STD)]	0.5 (± 2.3)	0.7 (± 3.0)	0.1 (± 0.7)	0.245	0.6 (± 2.5)	0.0 (± 0.0)	0.118
*S. aureus* detection [no. of patients (%)]	11 (10.6%)	2 (3.6%)	7 (16.7%)	0.028	10 (12.2%)	1 (4.5%)	0.300
*P. aeruginosa* detection [no. of patients (%)]	35 (33.7%)	31 (56.4%)	4 (9.5%)	< 0.001	22 (26.8%)	13 (59.1%)	0.004
*P. aeruginosa*-specific antibodies [normal/elevated, (% elevated)]							
against alkaline protease	70/14 (16.7%)	26/12 (31.6%)	38/1 (2.6%)	0.001	62/10 (13.9%)	8/4 (33.3%)	0.094
against elastase	69/15 (17.9%)	25/13 (34.2%)	38/1 (2.6%)	< 0.001	60/12 (16.7%)	9/3 (25.0%)	0.485
against exotoxin A	65/19 (22.6%)	24/14 (36.8%)	34/5 (12.8%)	0.015	58/14 (19.4%)	7/5 (41.7%)	0.088
WBC [× 10^3^/μl (± STD)]	9.1 (± 4.3)	10.1 (± 5.2)	7.5 (± 2.4)	0.002	8.8 (± 3.3)	10.1 (± 7.0)	0.891
CRP [mg/l (± STD)]	6.5 (± 19.1)	11.6 (± 25.1)	0.7 (± 1.0)	< 0.001	4.6 (± 12.2)	13.6 (± 33.6)	0.114
Total IgG [lowered/normal/elevated]	12/62/25	3/26/21	7/31/4	0.002	12/49/19	0/13/6	0.187
Total IgE level [normal/elevated, (% elevated)]	65/37 (36.3%)	27/26 (49.1%)	33/9 (21.4%)	0.006	54/27	11/10	0.225
Fatty acid-binding protein 2 [pg/ml (± STD)]	2407 (± 1471)	2279 (± 1429)	2565 (± 1524)	0.248	2540 (± 1574)	1889 (± 871)	0.115
FEV_1_ at serum sampling [% predicted (± STD)]	73.1 (± 24.1)	55.8 (± 15.9)	95.8 (± 9.9)	–	75.7 (± 23.9)	64.2 (± 23.1)	0.057
Cough frequency [none/temporarily/permanently/at night]	11/64/19/6	1/33/14/4	8/28/4/1	0.007	11/50/16/4	0/14/3/2	0.278
Number of pulmonary exacerbations within the last 12 months	0.56 (± 0.77)	0.76 (± 0.90)	0.40 (± 0.55)	0.044	0.52 (± 0.72)	0.74 (± 0.93)	0.293
Systemic antibiotic therapy at serum sampling [no. of patients (%)]	30 (28.8%)	19 (34.5%)	10 (23.8%)	0.252	23 (28.0%)	7 (31.8%)	0.729
Systemic antibiotic therapy courses (no. (± STD)							
12 month before serum sampling	1.6 (± 2.0)	2.2 (± 2.3)	0.9 (± 1.1)	< 0.001	1.4 (± 1.6)	2.2 (± 3.0)	0.477
6 month after serum sampling	1.0 (± 1.4)	1.3 (± 1.7)	0.5 (± 0.8)	0.008	0.8 (± 1.1)	1.5 (± 2.4)	0.314
Nebulized antibiotic [none/temporarily/permanently]							
12 month before serum sampling	58/16/30	18/9/28	33/7/2	< 0.001	54/11/17	4/5/13	< 0.001
at serum sampling	51/38	14/34	30/4	< 0.001	48/23	3/15	< 0.001
6 month after serum sampling	57/21/26	17/14/24	33/7/2	< 0.001	52/16/14	5/5/15	0.001
Inhaled corticosteroids [no. of patients (%)]	14 (14.6%)	9 (18.4%)	5 (12.5%)	0.449	9 (11.7%)	5 (26.3%)	0.106
Exocrine pancreatic insufficiency [no. of patients (%)]	93 (89.4%)	50 (90.9%)	39 (92.9%)	0.730	72 (87.8%)	21 (95.5%)	0.300
CF-related diabetes mellitus [no. of patients (%)]	11 (10.6%)	8 (14.5%)	3 (7.1%)	0.255	9 (11.0%)	2 (9.1%)	0.799
CF-related liver disease [no. of patients (%)]	30 (28.8%)	16 (29.1%)	14 (33.3%)	0.654	22 (27.8%)	8 (32.0%)	0.729

*FEV1* forced expiratory volume in 1 s, *BMI* body mass index, *STD* standard deviation, *BDG* (1→3)-β-D-glucan, *IQR* interquartile range, *GM* galactomannan, *ODI* optical density index, *WBC* white blood cell count, *CRP* C-reactive protein, *no* number, *CF* cystic fibrosis

**Table 2:** Patient demographics, clinical and microbiological results stratified after serum BDG and GM positivity

	All patients	Serum BDG level			Serum GM level		
	(n = 104)	Normal (< 60) (*n* = 77)	Elevated (≥60) (*n* = 27)	*P* value	Normal (< 0.5) (*n* = 97)	Elevated (≥0.5) (n = 7)	*P* value
Age [years (min-max)]	19.3 (4–51)	18.1 (4–48)	22.7 (4–51)	0.079	18.6 (4–51)	29.6 (14–51)	0.047
Sex [female/male]	43/61	31/46	12/15	0.704	40/57	3/4	0.933
BMI [kg/m^2^ (± STD)]	19.7 (± 3.9)	19.8 (± 3.9)	19.4 (± 3.9)	0.739	19.8 (± 3.9)	19.3 (± 2.7)	0.897
Mean BDG [pg/ml (± STD)]	50 (± 84)	16 (± 17)	148 (± 116)	–	50 (± 86)	57 (± 26)	0.044
Median BDG [pg/ml (IQR)]	22 (3–62)	11 (0–28)	108 (69–179)		20 (3–56)	59 (48–68)	
BDG [normal/elevated, (% elevated)]	77/27 (26.0%)	–	–	–	73/24 (24.7%)	4/3 (42.9%)	0.291
Mean GM [ODI (± STD)]	0.18 (± 0.22)	0.15 (± 0.17)	0.24 (± 0.32)	0.013	0.13 (± 0.1)	0.89 (± 0.4)	–
Median GM [ODI (IQR)]	0.10 (0.1–0.2)	0.10 (0.10)	0.10 (0.10)		0.10 (0.10)	0.70 (0.6–1.2)	
GM [normal/elevated, (% elevated)]	97/7 (6.7%)	73/4 (5.2%)	24/3 (11.1%)	0.291	–	–	–
Persistent *A. fumigatus* detection [no. of patients (%)]	22 (21.2%)	11 (14.3%)	11 (40.7%)	0.004	19 (19.6%)	3 (42.9%)	0.145
S. aureus colonization/infection [no. of patients (%)]	11 (10.6%)	10 (13.0%)	1 (3.7%)	0.177	11 (11.3%)	0 (0.0%)	0.346
P. aeruginosa colonization/infection [no. of patients (%)]	35 (33.7%)	23 (29.9%)	12 (44.4%)	0.168	30 (30.9%)	5 (71.4%)	0.029
*P. aeruginosa*-specific antibodies [normal/elevated, (% elevated)]							
against alkaline protease	70/14 (16.7%)	52/11 (17.5%)	18/3 (14.3%)	0.735	67/12 (15.2%)	3/2 (40.0%)	0.149
against elastase	69/15 (17.9%)	52/11 (17.5%)	17/4 (19.0%)	0.869	67/12 (15.2%)	2/3 (60.0%)	0.011
against exotoxin A	65/19 (22.6%)	48/15 (23.8%)	17/4 (19.0%)	0.651	65/14 (17.7%)	0/5 (100.0%)	< 0.001
*A. fumigatus*-specific antibodies [normal/elevated, (% elevated)]	100/4 (3.8%)	74/3 (3.9%)	26/1 (3.7%)	0.964	93/4 (4.1%)	7/0 (0.0%)	0.584
*A. fumigatus*-specific IgE level [kU/l, (± STD)]	3.8 (± 9.9)	3.6 (± 9.5)	4.4 (± 11.2)	0.777	4.0 (± 10.2)	1.8 (± 1.7)	0.051
rAsp f4-IgE level	0.5 (± 2.5)	0.2 (± 1.1)	1.3 (± 4.5)	0.768	0.5 (± 2.5)	0.3 (± 0.4)	0.048
rAsp f6-IgE level	0.5 (± 2.3)	0.2 (± 0.8)	1.2 (± 4.3)	0.930	0.5 (± 2.4)	0.0 (± 0.0)	0.391
WBC [×10^3^/μl (± STD)]	9.1 (± 4.3)	8.6 (± 3.3)	10.3 (± 6.3)	0.183	9.0 (± 4.5)	9.2 (± 2.3)	0.529
CRP [mg/l (± STD)]	6.5 (± 19.1)	4.5 (± 12.2)	12.1 (± 30.9)	0.125	6.5 (± 19.6)	6.6 (± 9.0)	0.506
Total IgG [lowered/normal/elevated]	12/62/25	9/49/17	3/13/8	0.553	12/58/22	0/4/3	0.392
Total IgE level [normal/elevated]	65/37	53/24	12/13	0.060	61/34	4/3	0.707
Fatty acid-binding protein 2 [pg/ml (± STD)]	2411 (± 1481)	2468 (± 1588)	2236 (± 1105)	0.712	2373 (± 1469)	2917 (± 1673)	0.392
FEV_1_ at serum sampling [% predicted (± STD)]	73.1 (± 24.1)	77.1 (±22.8)	61.6 (±25.5)	0.007	73.6 (± 24.7)	66.1 (± 14.1)	0.407
Cough frequency [none/temporarily/permanently/at night]	11/64/19/6	11/49/11/3	0/15/8/3	0.038	11/58/18/6	0/6/1/0	0.589
Number of pulmonary exacerbations within the last 12 months	0.56 (± 0.77)	0.46 (± 0.72)	0.85 (± 0.83)	0.008	0.57 (± 0.78)	0.43 (± 0.54)	0.762
Systemic antibiotic therapy at serum sampling [no. of patients (%)]	30 (28.8%)	18 (23.4%)	12 (44.4%)	0.038	28 (28.9%)	2 (28.6%)	0.987
Systemic antibiotic therapy courses (no. (± STD)							
12 month before serum sampling	1.6 (± 2.0)	1.4 (± 1.8)	2.0 (± 2.4)	0.415	1.5 (± 2.0)	2.1 (± 1.6)	0.120
6 month after serum sampling	1.0 (± 1.4)	0.8 (± 1.1)	1.5 (± 2.1)	0.047	1.0 (± 1.5)	1.0 (± 1.0)	0.619
Nebulized antibiotic [none/temporarily/permanently]							
12 month before serum sampling	58/16/30	49/10/18	9/6/12	0.024	56/16/25	2/0/5	0.032
at serum sampling	51/38	43/25	8/13	0.042	49/33	2/5	0.109
6 month after serum sampling	57/21/26	46/15/16	11/6/10	0.173	55/20/22	2/1/4	0.124
Inhaled corticosteroids [no. of patients (%)]	14 (14.6%)	10 (13.0%)	4 (14.8%)	0.662	13 (13.4%)	1 (14.3%)	0.982
Exocrine pancreatic insufficiency [no. of patients (%)]	93 (89.4%)	67 (87.0%)	26 (96.3%)	0.177	86 (88.7%)	7 (100%)	0.346
CF-related diabetes mellitus [no. of patients (%)]	11 (10.6%)	7 (9.1%)	4 (14.8%)	0.405	9 (9.3%)	2 (28.6%)	0.109
CF-related liver disease [no. of patients (%)]	30 (28.8%)	25 (32.5%)	5 (18.5%)	0.169	28 (28.9%)	2 (28.6%)	0.987

*BDG* (1→3)-β-D-glucan, *GM* galactomannan, *BMI* body mass index, *STD* standard deviation, *IQR* interquartile range, *ODI* optical density index, *WBC* white blood cell count, *CRP* C-reactive protein, *no* number, *FEV1* forced expiratory volume in 1 s, *CF* cystic fibrosis

**Table 3:** Correlation of FEV_1_ at serum sampling, BDG and GM with continuous clinical and microbiological parameters (Spearman-Rho test)

	FEV_1_ at serum sampling			BDG			GM		
	Correlation Coefficient r	Significance level *p*-value	Coefficient of determination r^2^	Correlation Coefficient r	Significance level *p*-value	Coefficient of determination r^2^	Correlation Coefficient r	Significance level p-value	Coefficient of determination r^2^
Age	−0.382	< 0.001	0.146	0.187	0.057	0.035	0.186	0.058	0.035
BMI	−0.028	0.787	0.001	- 0.075	0.452	0.006	−0.041	0.682	0.002
FEV_1_ at serum sampling	–	–	–	- 0.460	< 0.001	0.212	−0.213	0.037	0.045
No. of pulmonary exacerbations (last 12 month)	−0.301	0.004	0.091	0.266	0.009	0.071	0.024	0.818	0.001
Systemic antibiotic therapy courses									
12 month before serum sampling	−0.438	< 0.001	0.192	0.281	0.004	0.079	0.186	0.059	0.035
6 month after serum sampling	−0.338	0.001	0.114	0.277	0.005	0.077	0.232	0.019	0.054
WBC	−0.450	< 0.001	0.203	0.246	0.012	0.061	0.168	0.091	0.028
CRP	−0.621	< 0.001	0.386	0.267	0.006	0.071	0.131	0.188	0.017
BDG	−0.460	< 0.001	0.212	–	–	–	0.281	0.004	0.079
GM	− 0.213	0.037	0.045	0.281	0.004	0.079	–	–	–
*A. fumigatus*-specific IgE level	−0.311	0.002	0.097	0.181	0.070	0.033	0.062	0.154	0.004
rAsp f4-IgE level	−0.203	0.051	0.041	0.151	0.134	0.023	0.041	0.688	0.002
rAsp f6-IgE level	−0.066	0.532	0.004	0.051	0.613	0.003	−0.179	0.074	0.032
Fatty acid-binding protein 2	0.159	0.125	0.025	0.044	0.666	0.002	0.072	0.475	0.005

*FEV1* forced expiratory volume in 1 s, *BDG* (1→3)-β-D-glucan, *GM* galactomannan, *BMI* body mass index, *no* number, *WBC* white blood cell count, *CRP* C-reactive protein

### Microbiological predictors of an impaired FEV1_predicted_ value

Fifty-five patients (56.7%) of our CF cohort suffered from moderate to very severe airflow limitation (FEV1_predicted_ < 80%), whereas 42 patients (43.3%) had only mild or no airflow limitation (FEV1_predicted_ ≥ 80%). In the univariate analysis, the group of patients with a FEV1_predicted_ < 80% was significantly older (24.0 versus 15.7 years, *p* <  0.001), had a significantly higher serum concentration of BDG (68 versus 32 pg/ml, *p* = 0.003), but not of GM (0.22 versus 0.14 optical density index [ODI], *p* = 0.150), and contained a higher percentage of patients with an elevated BDG (34.5% versus 14.3%, *p* = 0.024). In addition, this group had higher levels of *A. fumigatus*-specific IgE and there was a trend towards a higher percentage of patients with persistent *A. fumigatus* detection (29.1 versus 14.3%, *p* = 0.084) (Table 1).

Patients with a FEV1_predicted_ < 80% were more often colonized by *P. aeruginosa* and less likely colonized by *S. aureus*. *Pseudomonas aeruginosa*-specific antibodies, WBC, CRP, total IgG and IgE, cough frequency, number of pulmonary exacerbations within the last 12 months and consumption of systemic and nebulized antibiotics before and after serum sampling were also significantly elevated in this group (Table 1).

To examine, which parameters are independent predictors of the lung function, we performed a multivariate analysis with FEV1_predicted_ as dependent variable and all parameters with a significant difference in the univariate analysis as independent variables. The results are shown in Table 4. BDG, WBC, *S. aureus-* and *P. aeruginosa-*colonization turned out to be independent predictors of the FEV1_predicted_ in the multivariate analysis. These parameters were able to explain 52.6% of the variation of FEV1_predicted_ which corresponds to a strong effect according to Cohen [11]. An increase of the BDG concentration of 10 pg/ml was associated with an average loss in FEV1_predicted_ of 1%.

**Table 4:** Multivariate analysis with FEV1_predicted_ at serum sampling as dependent variable

Independent variables	Regression coefficient	*p*-value	R^2^ corr.	Effect size f
Age	−0.073	0.772	52.6%	1.05 (strong effect)
BDG	−0.098	0.002		
Persistent *A. fumigatus* detection	−2.951	0.562		
*A. fumigatus*-specific IgE level	0.045	0.810		
*S. aureus* detection	16.337	0.011		
*P. aeruginosa* detection	−13.628	0.023		
WBC	−2.911	< 0.001		
CRP	−0.015	0.933		
Systemic antibiotic therapy within the last 12 month	− 1.939	0.115		
Nebulized antibiotic within the last 12 month	0.890	0.832		

*FEV1* forced expiratory volume in 1 s, *BDG* (1→3)-β-D-glucan, *WBC* white blood cell count, *CRP* C-reactive protein, *R*^*2*^ coefficient of determination

### Characteristics of patients positive for *A. fumigatus*

Analysis of fungal cultures revealed that 22 patients (21.2%) were persistently colonized/infected with *A. fumigatus*, whereas one patient was only transiently colonized and 81 patients (77.9%) were negative for *A. fumigatus* (Table1). In addition, *Candida* spp. were detected in 55 patients (52.9%), *Penicillium* spp. in three patients (2.9%), *Scedosporium* sp. in two patients (1.9%) and *Lomentospora prolificans, Exophiala dermatitidis* and *Trichosporon asahii* in one patient each.

**Fig. 2:**
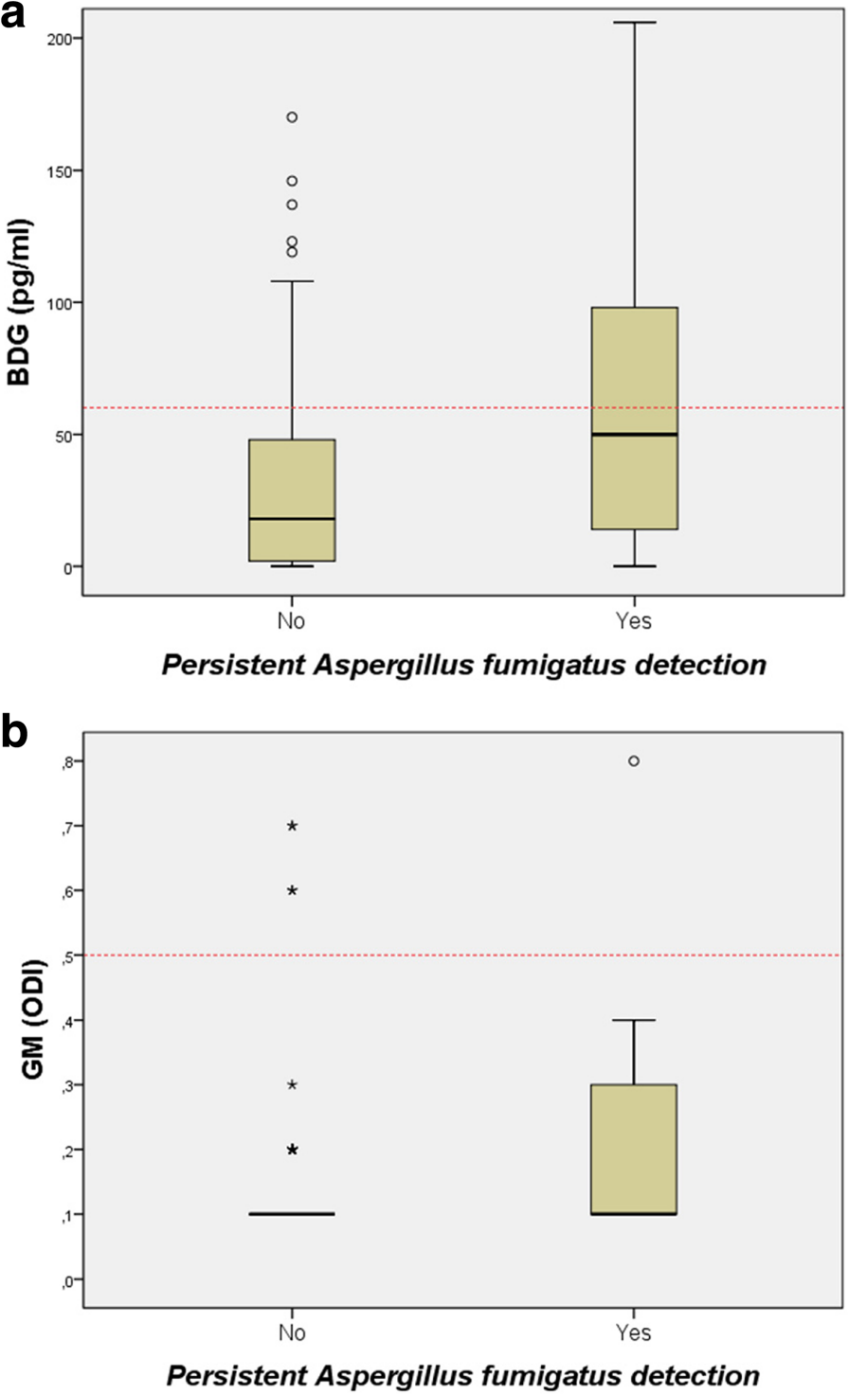
Box-Plots of BDG (**a**) and GM (**b**) results stratified by persistent *A. fumigatus* detection. The red dotted line indicates the manufacturer’s cut-off value for the biomarker. The small circles depict outliers and the stars extreme outliers. For better display not all extreme outliers are shown. BDG: (1→3)-β-D-glucan; GM: galactomannan

**Fig. 3:**
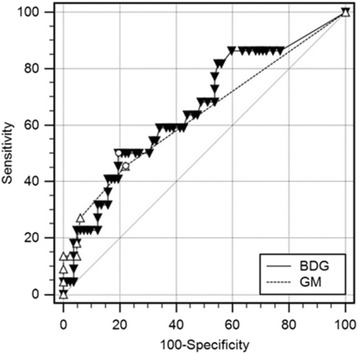
Receiver-operating-characteristic (ROC)-analysis for discrimination between *A. fumigatus*-positive and -negative patients. The cut-off with the highest Youden index (black circles) was ≥60 pg/ml for BDG (sensitivity 50.0%, specificity 80.5%) and > 0.1 ODI for GM (sensitivity 45.5%, specificity 78.0%). The area under the ROC-curve was not significantly different for BDG and GM (0.658 versus 0.635, *p* = 0.760). BDG: (1→3)-β-D-glucan; GM: galactomannan

The mean serum concentrations of BDG and GM in patients that were persistently positive for *A. fumigatus* were significantly higher (89 pg/ml and 0.30 ODI, respectively) than in patients without persistent growth of *A. fumigatus* (40 pg/ml [*p* = 0.022] and 0.15 ODI [*p* = 0.013], respectively) (Fig. 2). Although the difference in BDG concentrations between the two subgroups was small, there were significantly more patients with elevated BDG levels (≥ 60 pg/ml) in the *A. fumigatus*-positive subgroup (50.0 versus 19.5%, *p* = 0.004) (Table 1). ROC-analysis for discrimination between *A. fumigatus*-positive and -negative patients gave an area under the ROC curve (AUC) of 0.658 (95%-CI: 0.559–0.748) for BDG and 0.635 (0.535–0.727) for GM (Fig. 3). The AUC of BDG and GM was not significantly different (*p* = 0.760). The cut-off with the highest Youden index was ≥60 pg/ml for BDG (sensitivity 50.0%, specificity 80.5%) and > 0.1 ODI for GM (sensitivity 45.5%, specificity 78.0%). In addition, patients with persistent *A. fumigatus* detection were significantly older (24.8 versus 17.9 years, *p* = 0.001), had a higher BMI (21.6 versus 19.2 kg/m^2^, *p* = 0.006) and were more often colonized with *P. aeruginosa* (59.1 versus 26.8%, 0.004) (Table 1). The FEV1_predicted_ was lower in patients with a positive culture for *A. fumigatus* (64.2 versus 75.7%, *p* = 0.057) and they were treated more frequently with nebulized antibiotics in the year before, at and during the 6 months after serum sampling. Although the frequency of systemic antibiotic therapy was also higher in the *A. fumigatus*-positive subgroup, these differences were not statistically significant. Interestingly, none of the *Aspergillus*-specific antibodies was significantly elevated in *A. fumigatus*-positive patients (Table 1).

### Characteristics of patients with elevated BDG and GM serum concentrations

To further investigate possible differences in the diagnostic value of fungal cultures versus biomarker measurements, we also analyzed the association of BDG and GM with clinical parameters. Of our CF cohort, 27 patients (26.0%) had elevated BDG levels (median 108 pg/ml, IQR 69–179) and 7 patients (6.7%) elevated GM levels (median ODI 0.7, IQR 0.6–1.2). Patients with an elevated serum BDG (≥ 60 pg/ml) had a significantly higher serum GM (0.24 versus 0.15, *p* = 0.013) than patients with a normal BDG and vice versa (*p* = 0.044) (Table 2). BDG and GM levels showed a significant correlation but with a very weak effect size (Table 3).

Patients with an elevated serum BDG were more frequently *A. fumigatus*-positive (40.7 versus 14.3%, *p* = 0.004). They had a significantly lower FEV1_predicted_ than patients with a normal BDG (61.6 versus 77.1%, *p* = 0.007) and BDG levels correlated inversely with the paired FEV1_predicted_ (Table 4). Furthermore, patients with an elevated BDG tended to be older (22.7 versus 18.1 years, *p* = 0.079), had a significantly higher cough rate and more pulmonary exacerbations during the last year. They had received more frequently a nebulized antibiotic, had a higher probability of systemic and nebulized antibiotic therapy at serum sampling and were treated more often with systemic antibiotics during the 6 months follow-up (Table 2).

Patients with elevated GM levels (> 0.5 ODI) were also older and received more often nebulized antibiotics during the last year. However, in contrast to BDG, patients with an elevated serum concentration of GM showed higher levels of *A. fumigatus*-specific IgE and significantly lower levels of rAsp f4-IgE. Interestingly, patients with an elevated GM were more frequently colonized/infected with *P. aeruginosa* and showed more often elevated antibody levels against *P. aeruginosa* elastase and exotoxin A (Table 2).

Most importantly, patients with elevated GM levels did not have significantly reduced FEV1_predicted_ values (66.1% versus 73.6%, *p* = 0.407). However, this was only the case if the cut-off value was used that is recommended for the diagnosis of invasive fungal disease (ODI ≥ 0.5). After applying the cut-off calculated by our ROC-analysis (ODI > 0.1), patients with elevated GM levels (*n* = 18) showed significantly reduced FEV1_predicted_ values (65.4% versus 76.1%, *p* = 0.042).

## Discussion

*Aspergillus fumigatus* is cultured from respiratory samples of CF patients with a prevalence of 6–57% [2]. There is an ongoing debate whether its presence is contributing to the decline of pulmonary function in these patients [12]. If this were the case, it would have far-reaching implications because CF patients might profit from antifungal therapy.

In our cohort, 21.2% of patients were persistently colonized or infected with *A. fumigatus*. Univariate analysis revealed that these patients were in a more advanced stage of disease, i.e. they were older, had a higher BMI, were more often colonized or infected with *P. aeruginosa*, were treated more frequently with nebulized but not systemic antibiotics and, most importantly, had a lower FEV1_predicted_ at serum sampling. Similar findings concerning the association between culture positivity for *Aspergillus* and increasing age [13–15], lower BMI [13], detection of *P. aeruginosa* [13, 16], exposure to nebulized antibiotics [13, 14, 17–19] and reduced FEV1 [3, 4, 13] were reported in the past. However, there are also studies that could not find such an association between the detection of *Aspergillus* spp. and lung function [18, 20]. So far, the reasons for these conflicting results are unclear.

In the present study, we performed for the first time a detailed correlative analysis of BDG and GM antigenemia, colonization/infection with *A. fumigatus* and pulmonary function in CF patients. Our data shows that BDG and GM were significantly higher in *Aspergillus*-positive compared to *Aspergillus*-negative patients (BDG 89 versus 40 pg/ml and GM 0.3 versus 0.15 ODI, respectively). These concentrations are lower than the BDG and GM levels that have been observed in patients with invasive pulmonary aspergillosis (186 pg/ml and 0.70 ODI, respectively) [21]. Accordingly, most of our patients with *Aspergillus* detected in their airways would not fall into the category “serum antigen positive” if the cut-off values for invasive disease were applied (≥ 80 pg/ml and ≥ 0.5 ODI, respectively). However, it seems plausible that in CF patients with chronic *Aspergillus* colonization/infection less BDG or GM is released into the blood as compared to severely immunocompromised patients with angioinvasive disease. This is supported by our ROC analysis which gave optimal BDG and GM cut-off values for discrimination between *Aspergillus*-positive and *Aspergillus*-negative patients of ≥60 pg/ml and > 0.1 ODI, respectively. After applying these cut-offs to our study population, there were significantly more patients with elevated BDG and GM levels in the *Aspergillus*-positive than in the *Aspergillus*-negative subgroup. Interestingly, in case of BDG there is an “indeterminate” range between 60 and 80 pg/ml defined by the manufacturer and patients without invasive fungal disease have BDG-levels below 60 pg/ml. Thus, it seems that CF-patients with persistent *Aspergillus* detection have BDG and GM levels that range between healthy and invasively infected patients.

The diagnostic performance of BDG and GM to distinguish between *Aspergillus*-positive and *Aspergillus*-negative patients was, despite the optimized cut-offs, rather low. The sensitivity and specificity were around 50% or 80%, respectively, for both biomarkers. This means that half the patients with respiratory cultures positive for *Aspergillus* did not have elevated biomarker levels. However, it also means that 80% of patients with elevated biomarker levels were *Aspergillus*-positive. This constellation of low sensitivity and high specificity could be explained by the existence of two subgroups within the *Aspergillus*-positive patients. One subgroup consists of patients that are merely colonized with *A. fumigatus* (exhibiting normal biomarker levels) and the other of patients with true *A. fumigatus* infection (exhibiting elevated biomarker levels). If this is the case, BDG and GM could potentially differentiate between these two subgroups. If one further assumes that only infected but not colonized patients will develop an *Aspergillus*-associated impairment of their lung function, it would imply that biomarkers are superior to culture in predicting the FEV1 evolution.

The analysis of the FEV1 in our CF population supports this hypothesis. In the univariate analysis BDG and GM levels are correlating inversely with the FEV1 and patients with elevated BDG or GM levels have significantly lower FEV1 values. Moreover, the multivariate analysis revealed that BDG, but not the culture of *A. fumigatus,* proved to be an independent predictor of the FEV1. These data also offer an explanation why previous studies reported inconsistent results concerning the correlation between *Aspergillus* detection and lung function decline.

Data on fungal biomarker levels in sera of CF patients with proven colonization/infection with *Aspergillus* are scarce. Recently, Rautemaa et al. [22] reported on the only study examining BDG levels in CF patients. They analyzed serum samples from an adult CF cohort (*n* = 46) at the time of stable disease and during pulmonary exacerbation. There was no significant difference in the BDG levels of patients with stable versus exacerbated disease (40.2 versus 48.7 pg/ml, *p* = 0.544) or between patients with versus without positive fungal culture (76 versus 37 pg/ml, *p* = 0.227). Unfortunately, the authors did not specify the fungi they detected. According to a personal communication by V. Rauteema (University of Manchester, UK), the cultured fungi were predominantly *Candida* spp., whereas only few *Aspergillus* spp. were isolated. Separate statistical analysis of both genera also did not show significant differences in BDG levels. However, the low number of patients with cultures positive for *Aspergillus* could explain why the BDG levels between *Aspergillus*-positive and -negative patients were not significantly different in their study.

Furthermore, Rautemaa et al. [22] reported that multiple serum samples collected over a period of 13 months from 6 patients showed that BDG-positive patients stayed positive and BDG-negative patients remained negative. This would support our hypothesis that BDG-antigenemia in CF patients is not a transient phenomenon but rather the consequence of a persistent condition like chronic *Aspergillus* infection.

Interestingly, Rautemaa et al. [22] found significantly elevated BDG-levels in patients with exocrine pancreatic insufficiency (PI) and CF-related diabetes (CFRD) and hypothesized that the chronically inflamed intestinal epithelium in CF could allow a translocation of BDG from the gut into the blood. However, we were not able to confirm this data. BDG levels were independent of PI and CFRD (data not shown). To further examine the gut as possible source of serum BDG, we measured intestinal fatty acid binding protein 2 (FABP2), a specific marker for gut mucosal injury [23–25], in the serum of our CF population. FABP2 was not elevated in patients with positive BDG levels arguing against an intestinal source of BDG.

So far, the only study on GM and the detection of *Aspergillus* in CF patients was performed by Warren et al. [26]. They analyzed GM levels from 138 CF patients. 43% of patients were persistently and 23% transiently colonized/infected with *Aspergillus*, whereas 34% of the patients were negative for *Aspergillus*. The GM levels of these three groups were identical and none of the patients had GM levels above an ODI of 0.5. These results are contradictory to our findings. Comparing the findings by Warren et al. [26] with our data*,* it is striking that 43% and 23% of their patients were persistently or intermittently culture-positive for *Aspergillus*, whereas in our cohort only 21% of patients were persistently and 1% were transiently *Aspergillus*-positive. This suggests that there were either profound differences in the study populations or in the diagnostic methodology. Patient recruitment and the utilized GM assay were identical. Unfortunately, the authors did not specify their fungal culture methodology. Also, the definitions for persistent and transient detection of *Aspergillus* were different. Warren et al. [26] defined ≥2 positive *Aspergillus* cultures in the last year as persistent colonization/infection, whereas we required > 50% positive cultures in the past 2 years. Essentially, this could mean that our patients were more heavily colonized/infected and therefore had higher GM levels. Interestingly, in the study by Warren et al. [26], patients colonized with *Aspergillus* were significantly younger than patients without. Again, this finding contrasts with our result (colonized patients were older), which is in accordance with other data showing that higher age is as a risk factor for *Aspergillus* colonization [13, 15].

Our study has some limitations. The retrospective and cross-sectional design leads to difficulties in distinguishing between cause and effect. It is currently unclear, whether colonization/infection with *Aspergillus* leads to a more rapid decline in lung function or whether patients with more severe disease exhibit an increased susceptibility to *Aspergillus*. Furthermore, we did not use culture media selective for *Scedosporium* spp.. Consequently, the number of patients with *Scedosporium* spp. present in respiratory samples might be underestimated. Since *Scedosporium* spp. also produce BDG, we cannot exclude the possibility that part of the BDG detected in the patients´ sera was derived from fungi other than *A. fumigatus*. Finally, it has been shown that the overall sensitivity of fungal culture is rather poor and that sputum GM or nucleic acid amplification techniques (NAT) are able to detect *Aspergillus* DNA in a considerable number of samples that were culture-negative [27]. In this context it seems plausible that patients with a negative *Aspergillus* culture but a positive sputum GM or NAT test are less heavily colonized than culture-positive patients. This would implicate that most *Aspergillus*-positive patients of our study were heavily colonized and that we might have missed the low-level colonized patients. Further research has to investigate whether these patients are serum BDG and GM positive and if low-level colonization will have an effect on pulmonary function. Therefore, prospective and longitudinal studies using sputum GM and NAT-assisted *Aspergillus* detection together with serum biomarker measurement are necessary to confirm and expand our data.

## Conclusions

We have shown that serum BDG and GM levels are significantly elevated in CF patients with persistent detection of *A. fumigatus* in respiratory specimens. BDG-, GM- and *Aspergillus*-positive patients were in a more advanced stage of disease. However, only BDG proved to be an independent predictor of the FEV1. Our data suggest that the serum level of BDG rather than the level of GM or the detection of *Aspergillus* by culture, might allow to predict the decline of lung function in CF and to identify patients that would profit from antifungal therapy.

## Abbreviations

AUC: Area under the ROC curve
BDG: (1→3)-β-D-glucan
BMI: Body mass index
CF: Cystic fibrosis
CFLD: CF-related liver disease
CFRD: CF-related Diabetes mellitus
CFTR: Cystic fibrosis transmembrane conductance regulator
CRP: C-reactive protein
FABP2: Fatty acid binding protein 2
FEV1: Forced expiratory volume in 1 s
GM: Galactomannan
GOLD: Global Initiative for Chronic Obstructive Lung Disease
Ig: Immunoglobulin
IQR: Interquartile range
MALDI-TOF-MS: Matrix Assisted Laser Desorption Ionization-Time of Flight Mass Spectrometry
NAT: Nucleic acid amplification techniques
ODI: Optical density index
PI: Exocrine pancreatic insufficiency
ROC: Receiver-operating-characteristic
SD: Standard deviation
WBC: Leucocyte count
